# Supplementary material for: Cellular underpinnings of the selective vulnerability to tauopathic insults in Alzheimer’s disease
Source: bioRxiv. 2023 Nov 29:2023.07.06.548027. Preprint. [Version 3] doi: 10.1101/2023.07.06.548027 (PMC10705232; doi:10.1101/2023.07.06.548027)
Supplement: Supplement 1 [file media-1.pdf]

Supplement to Cell Type Vulnerability

Justin Torok[1], Chaitali Anand[1], Pedro D. Maia[2], and Ashish Raj[1,*]

[1]University of California, San Francisco, Department of Radiology, San Francisco, CA, 94143, United States
[2]University of Texas at Arlington, Department of Mathematics, Arlington, TX, 76019, United States
[*]Address correspondence to ashish.raj@ucsf.edu

Supplemental Figures

Figure S1: Correlation structure of the Yao, *et al.* cell types. A. Heat map of the Pearson correlations of the gene expression profiles of the Yao, *et al.* [1] cell types. **B.** Heat map of the Pearson correlations of the regional distributions of the Yao, *et al.* cell types as inferred by MISS [2].

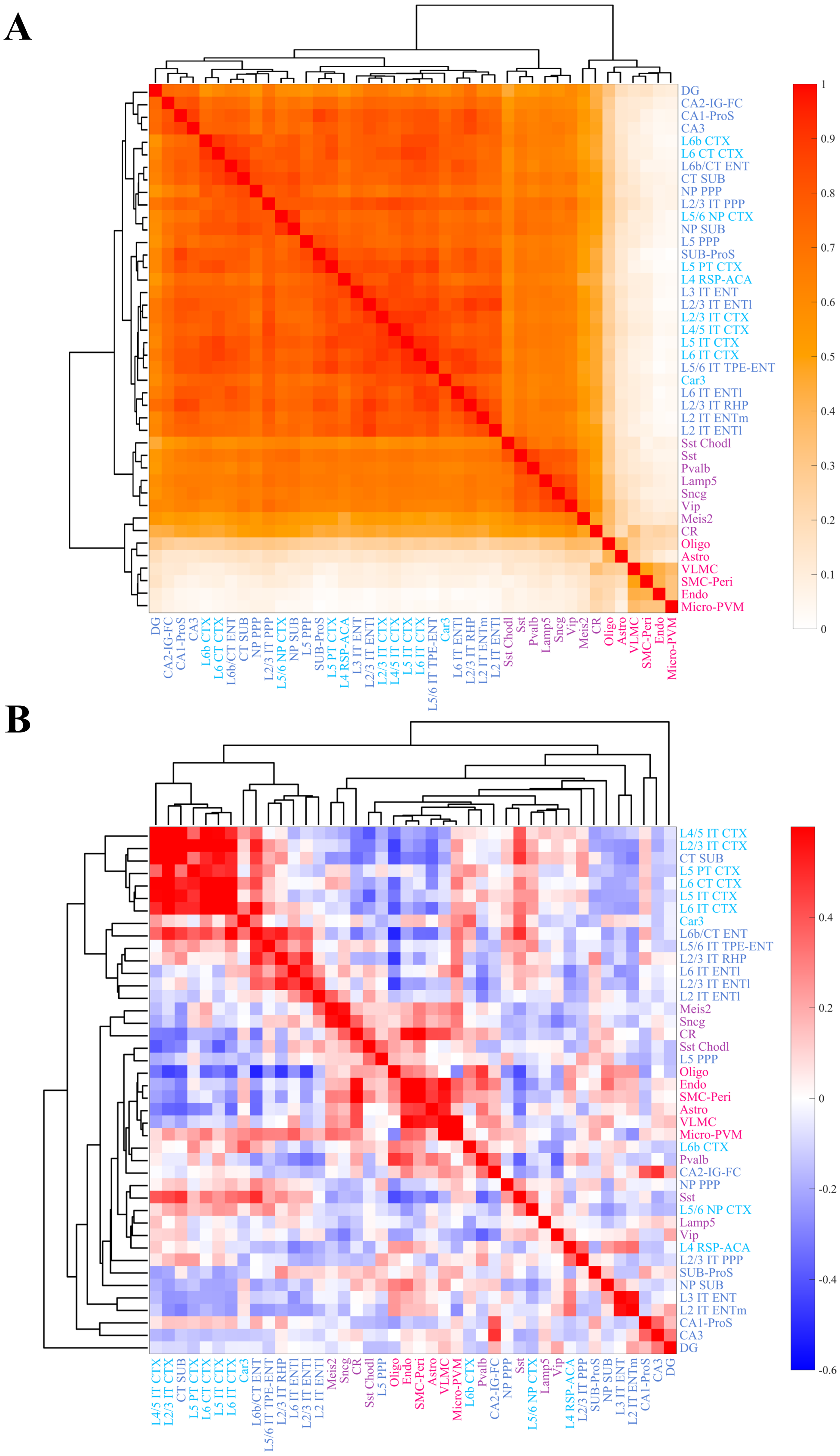
A
DG
CA2-IG-FC
CA1-ProS
CA3
L6b CTX
L6 CT CTX
L6b/CT ENT
CT SUB
NP PPP
L2/3 IT PPP
L5/6 NP CTX
NP SUB
L5 PPP
SUB-ProS
L5 PT CTX
L4 RSP-ACA
L3 IT ENT
L2/3 IT ENTl
L2/3 IT CTX
L4/5 IT CTX
L5 IT CTX
L6 IT CTX
L5/6 IT TPE-ENT
Car3
L6 IT ENTl
L2/3 IT RHP
L2 IT ENTm
L2 IT ENTl
Sst Chodl
Sst
Pvalb
Lamp5
Sncg
Vip
Meis2
CR
Oligo
Astro
VLMC
SMC-Peri
Endo
Micro-PVM
1
0.9
0.8
0.7
0.6
0.5
0.4
0.3
0.2
0.1
0
B
L4/5 IT CTX
L2/3 IT CTX
CT SUB
L5 PT CTX
L6 CT CTX
L5 IT CTX
L6 IT CTX
Car3
L6b/CT ENT
L5/6 IT TPE-ENT
L2/3 IT RHP
L6 IT ENTl
L2/3 IT ENTl
L2 IT ENTl
Meis2
Sncg
CR
Sst Chodl
L5 PPP
Oligo
Endo
SMC-Peri
Astro
VLMC
Micro-PVM
L6b CTX
Pvalb
CA2-IG-FC
NP PPP
Sst
L5/6 NP CTX
Lamp5
Vip
L4 RSP-ACA
L2/3 IT PPP
SUB-ProS
NP SUB
L3 IT ENT
L2 IT ENTm
CA1-ProS
CA3
DG
0.4
0.2
0
-0.2
-0.4
-0.6

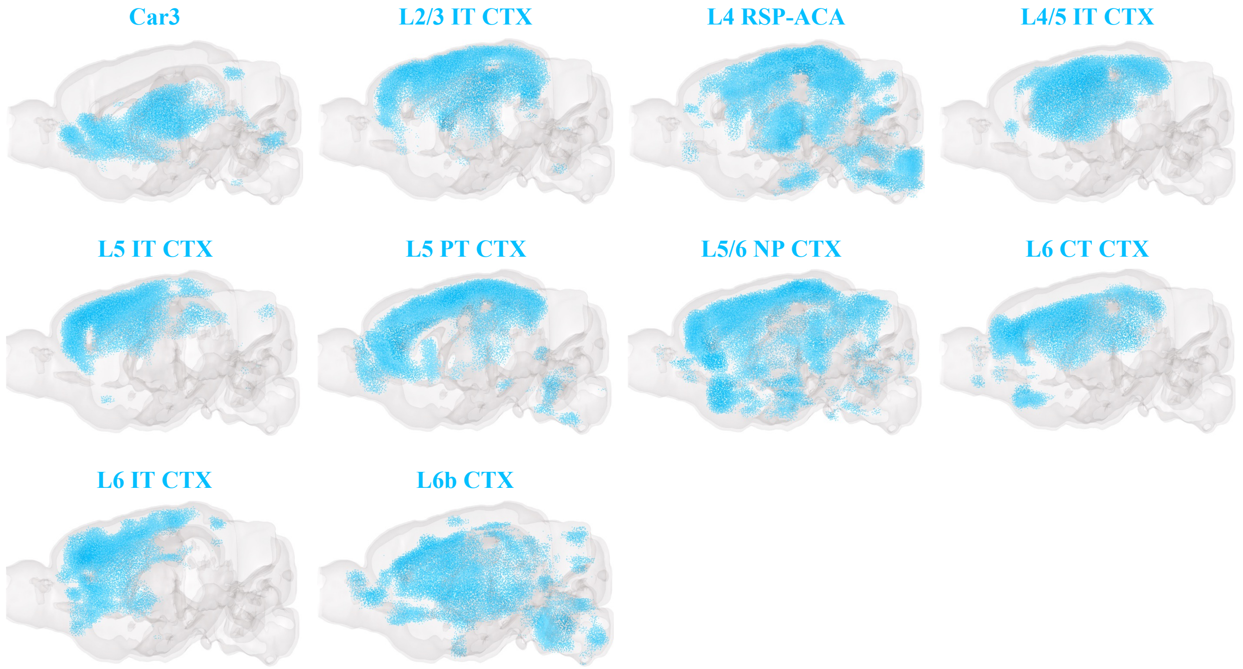

Figure S2: Distributions of cortical glutamatergic neurons. Sagittal views of the three-dimensional reconstructions of brain-wide densities of the cortical glutamatergic neurons in the Yao, *et al.* dataset [1]. Refer to **Table S1** and the original manuscript for further details on these cell types.

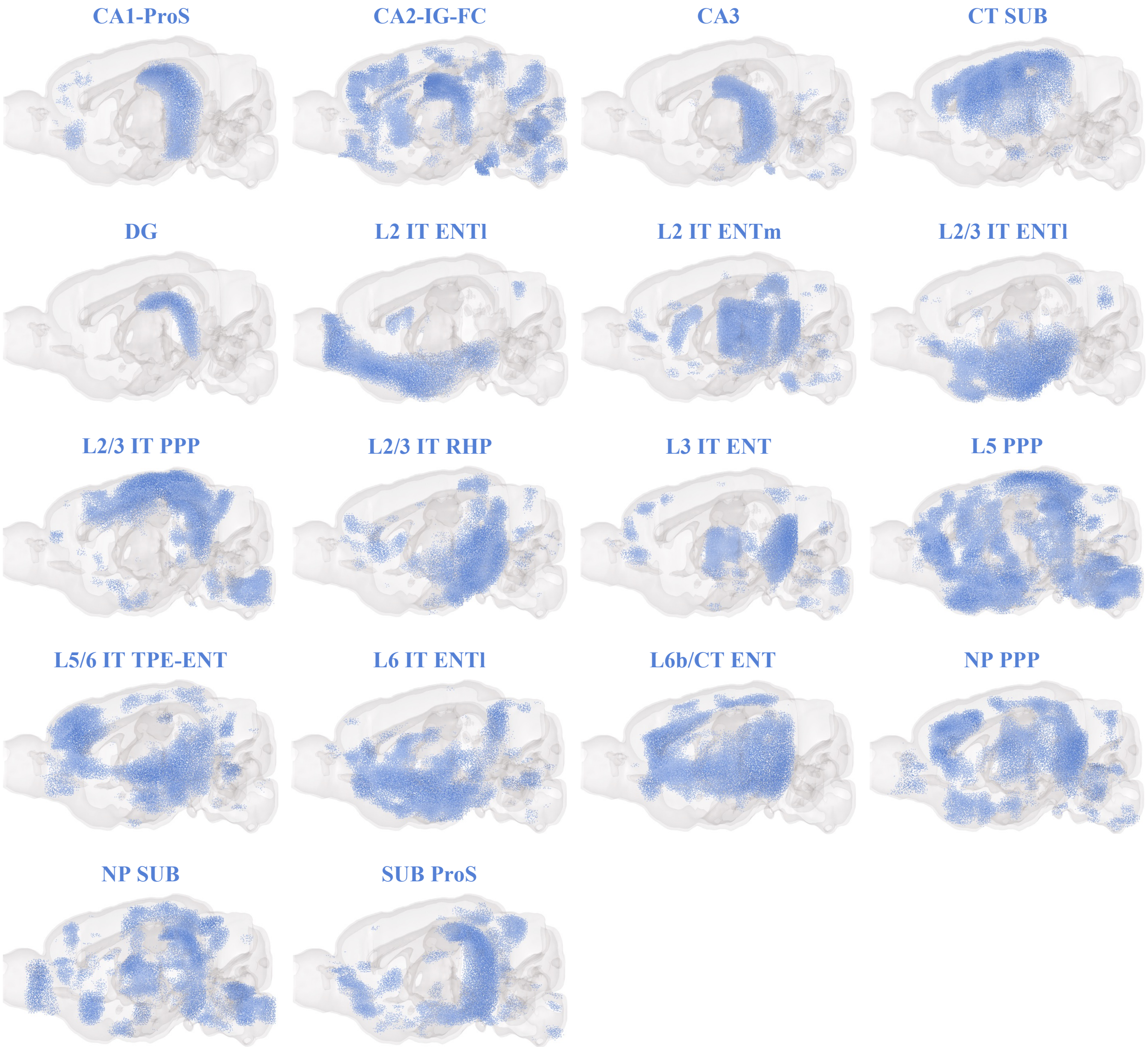

Figure S3: Distributions of hippocampal glutamatergic neurons. Sagittal views of the three-dimensional reconstructions of brain-wide densities of the hippocampal glutamatergic neurons in the Yao, *et al.* dataset [1]. Refer to **Table S1** and the original manuscript for further details on these cell types.

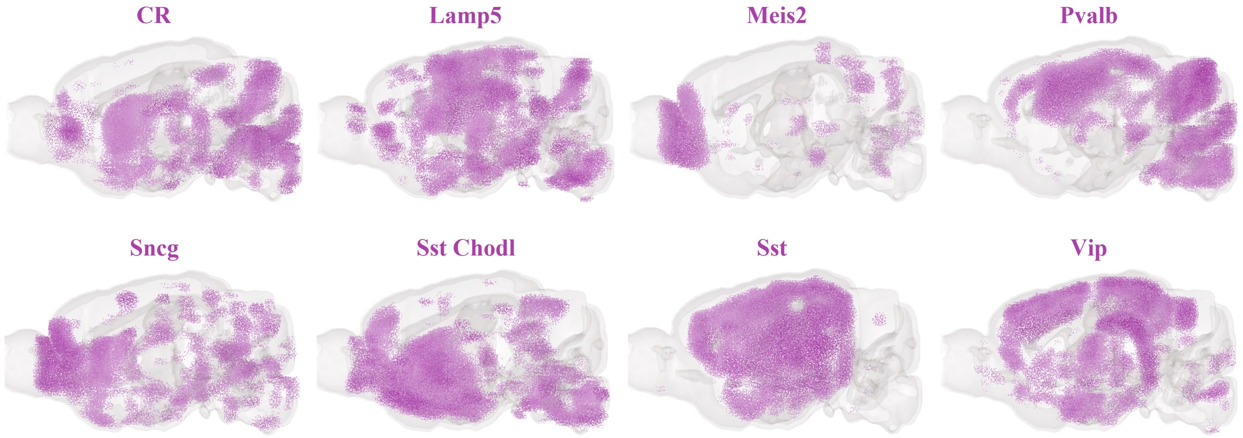

Figure S4: Distributions of GABAergic neurons. Sagittal views of the three-dimensional reconstructions of brain-wide densities of the GABAergic neurons in the Yao, *et al.* dataset [1]. Refer to **Table S2** and the original manuscript for further details on these cell types.

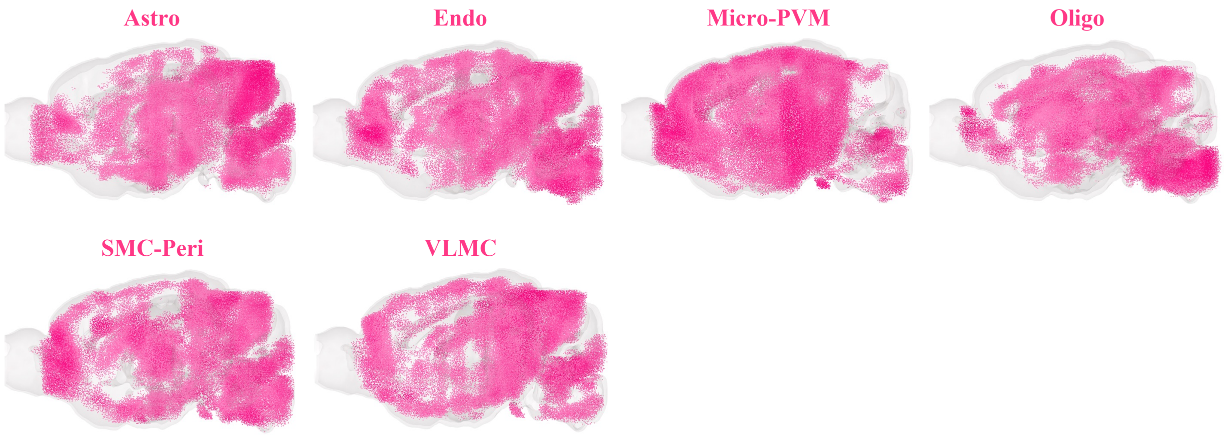

Figure S5: Distributions of non-neuronal cells. Sagittal views of the three-dimensional reconstructions of brain-wide densities of the non-neuronal cell types in the Yao, *et al.* dataset [1]. Refer to **Table S2** and the original manuscript for further details on these cell types.

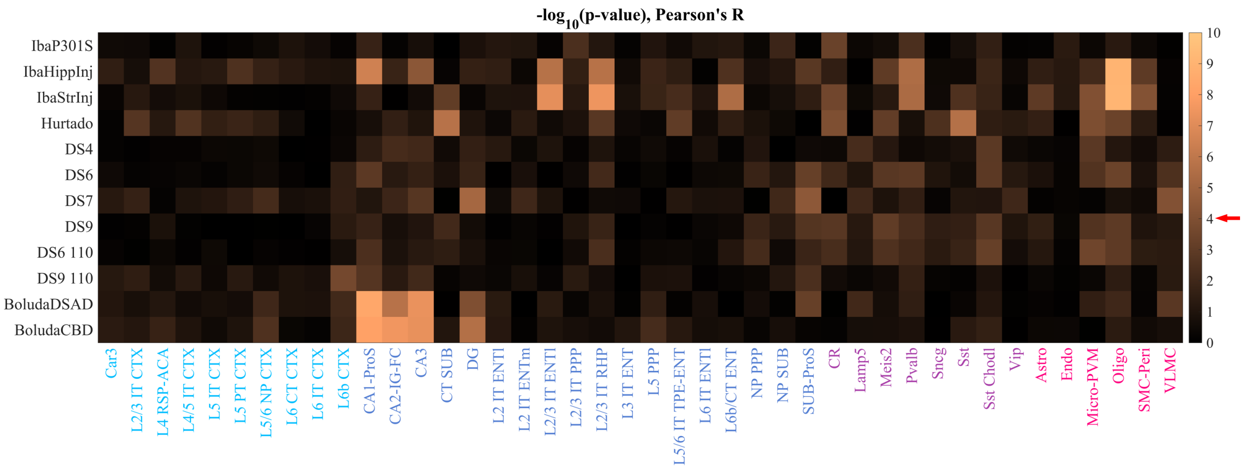

Figure S6: Statistical significance of the correlations in Figure 2. Heat map of the nominal -$\log_{10}$(p) values for the correlations presented in **Figure 2A**. The critical value corresponding to a Bonferroni-corrected significance level of 0.05 is -4.0 (red arrow).

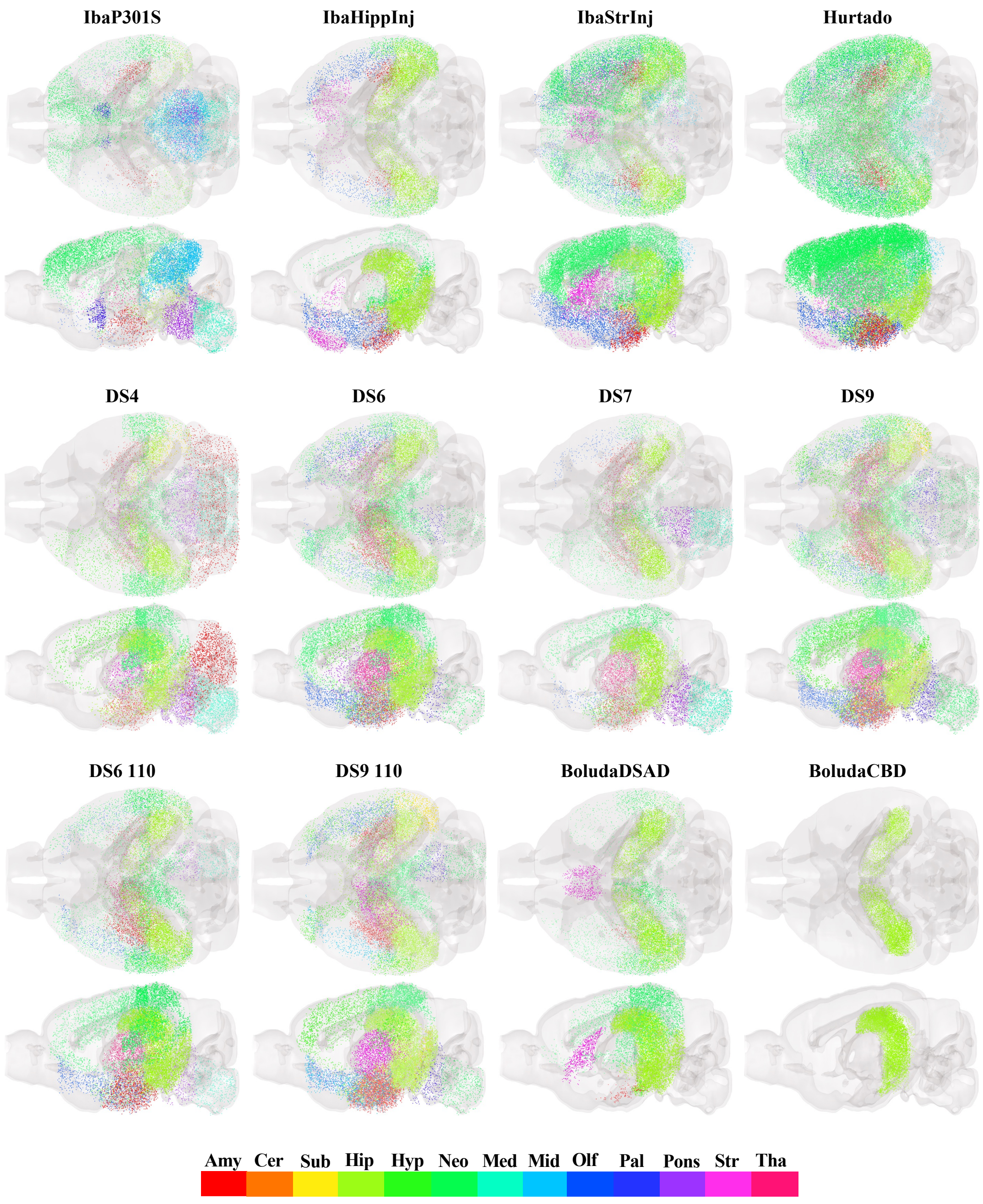

Figure S7: End-timepoint pathology glass brains. End-timepoint pathology for each of the twelve mouse tauopathy datasets, plotted in axial and sagittal views. See **Table S3** for descriptions of these datasets.

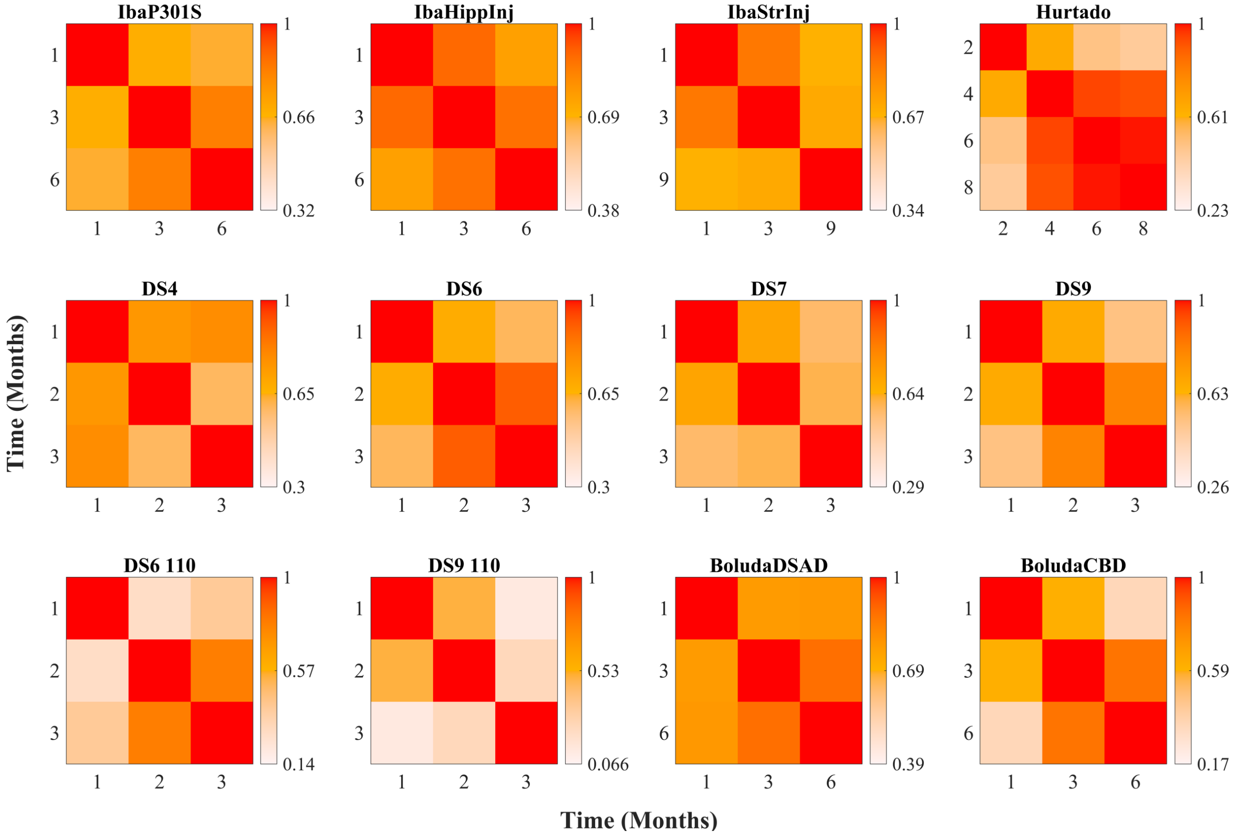

Figure S8: Correlation structure of the mouse tauopathy datasets Heat maps of the Pearson correlations between time points of the nine mouse tauopathy experiments analyzed in this study [3, 4, 5, 6, 7].

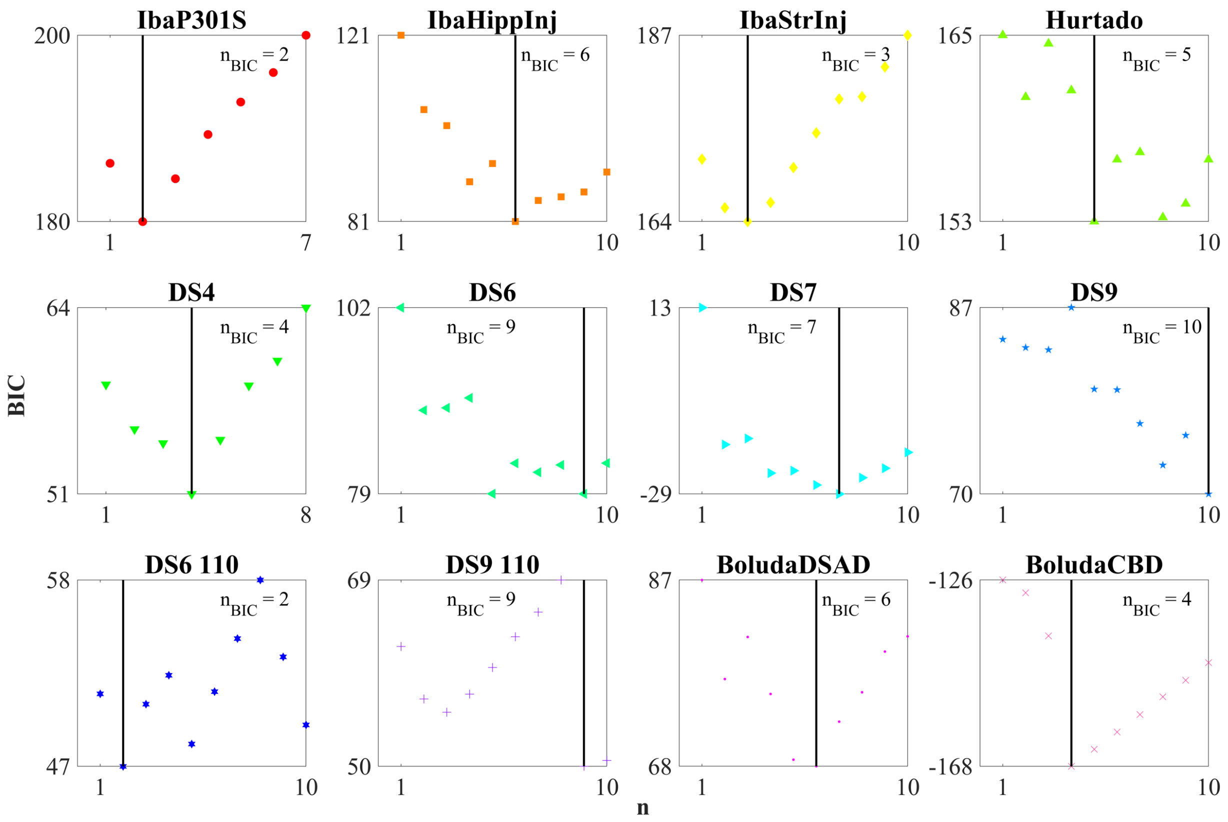

Figure S9: BIC plots for the multivariate linear models in Figure 3. Scatter plots of the BIC criterion with respect to the number of cell types added to the model (n) to determine the optimal sets for each tauopathy dataset.

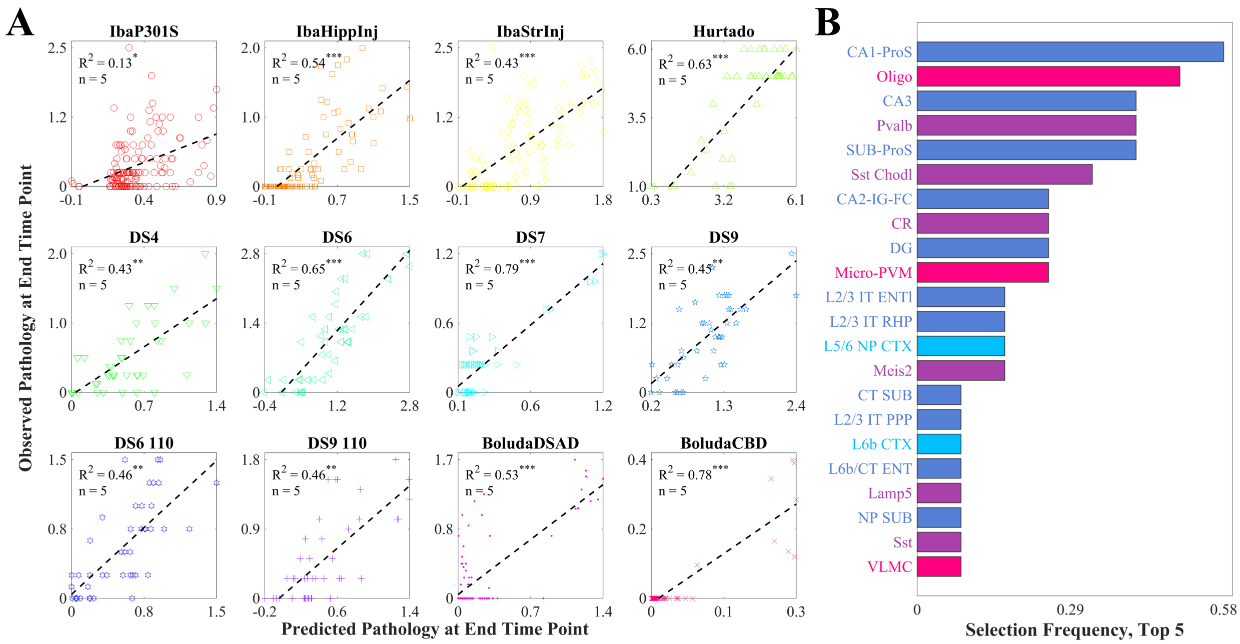

Figure S10: Multivariate analysis of end-timepoint pathology, top five cell types A. Scatter plots of the optimal cell-type-based models of tau pathology at the end time points for each of the nine mouse tauopathy studies, along with their associated R^2 values and the 5 cell types with the highest correlations to end-timepoint pathology (See **Figure 2**). **B.** Bar plot of the frequency with which cell types were included in the linear models in **A**. Of the 42 cell types in the Yao, *et al.* dataset, 22 were selected at least once.

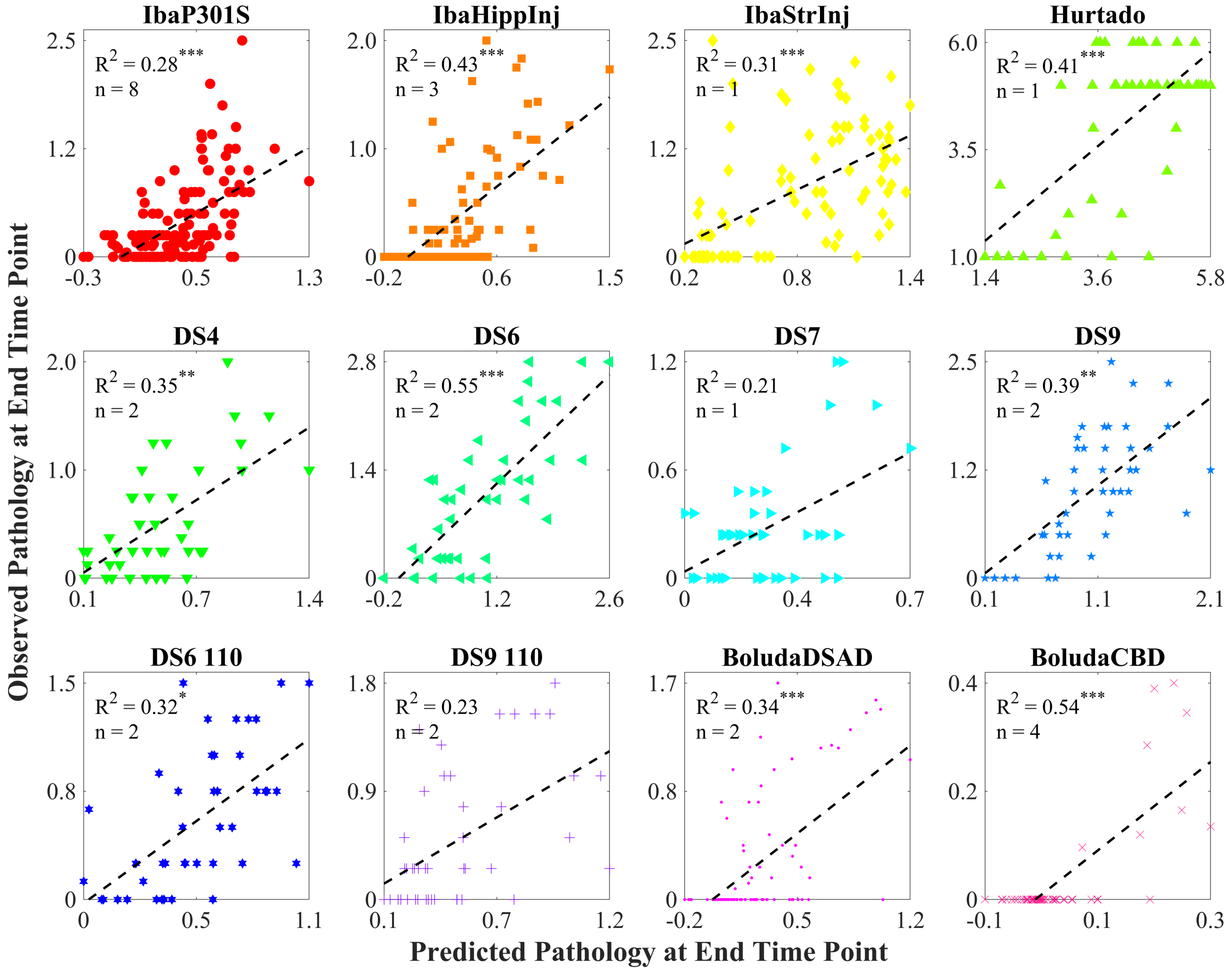

Figure S11: Multivariate analysis of end-timepoint pathology, AD genes (BIC). Scatter plots of the optimal cell-type-based models of tau pathology at the end time points for each of the nine mouse tauopathy studies, along with their associated R^2 values and the BIC-selected genes.

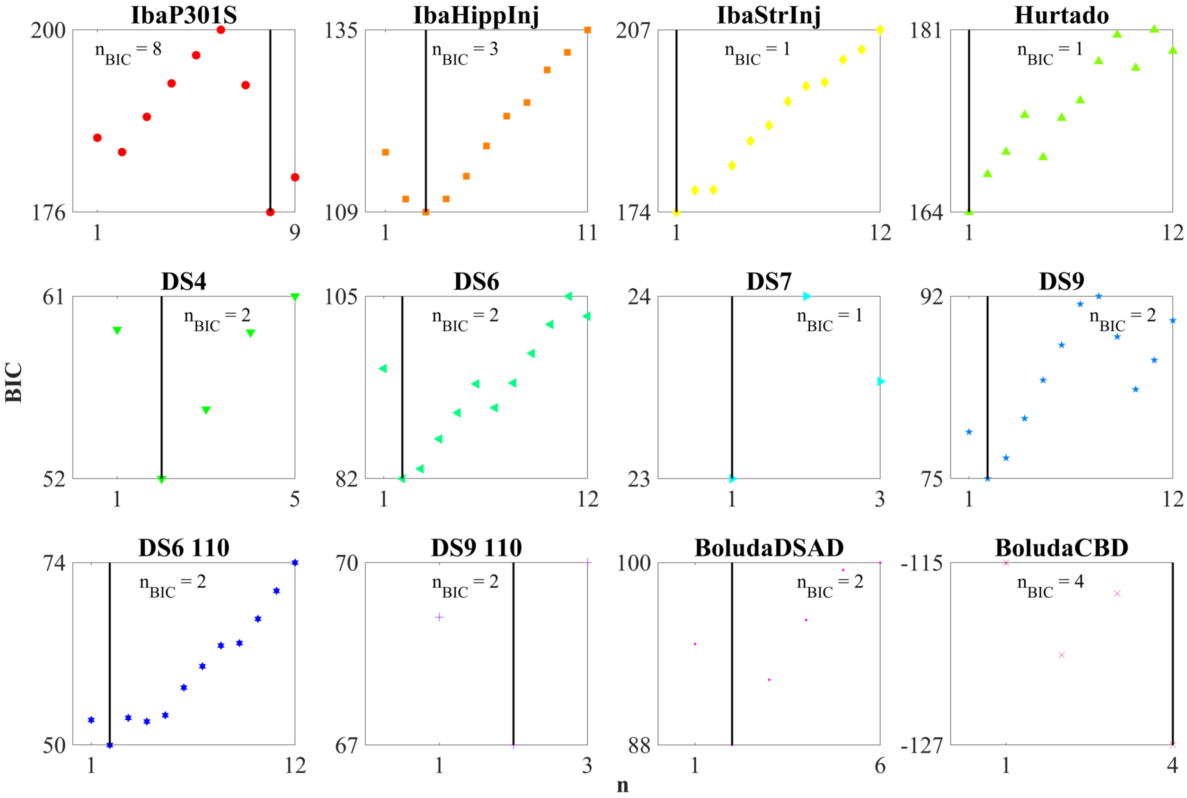

Figure S12: BIC plots for the multivariate linear models in Figure S11. Scatter plots of the BIC criterion with respect to the number of cell types added to the model (n) to determine the optimal sets for each tauopathy dataset.

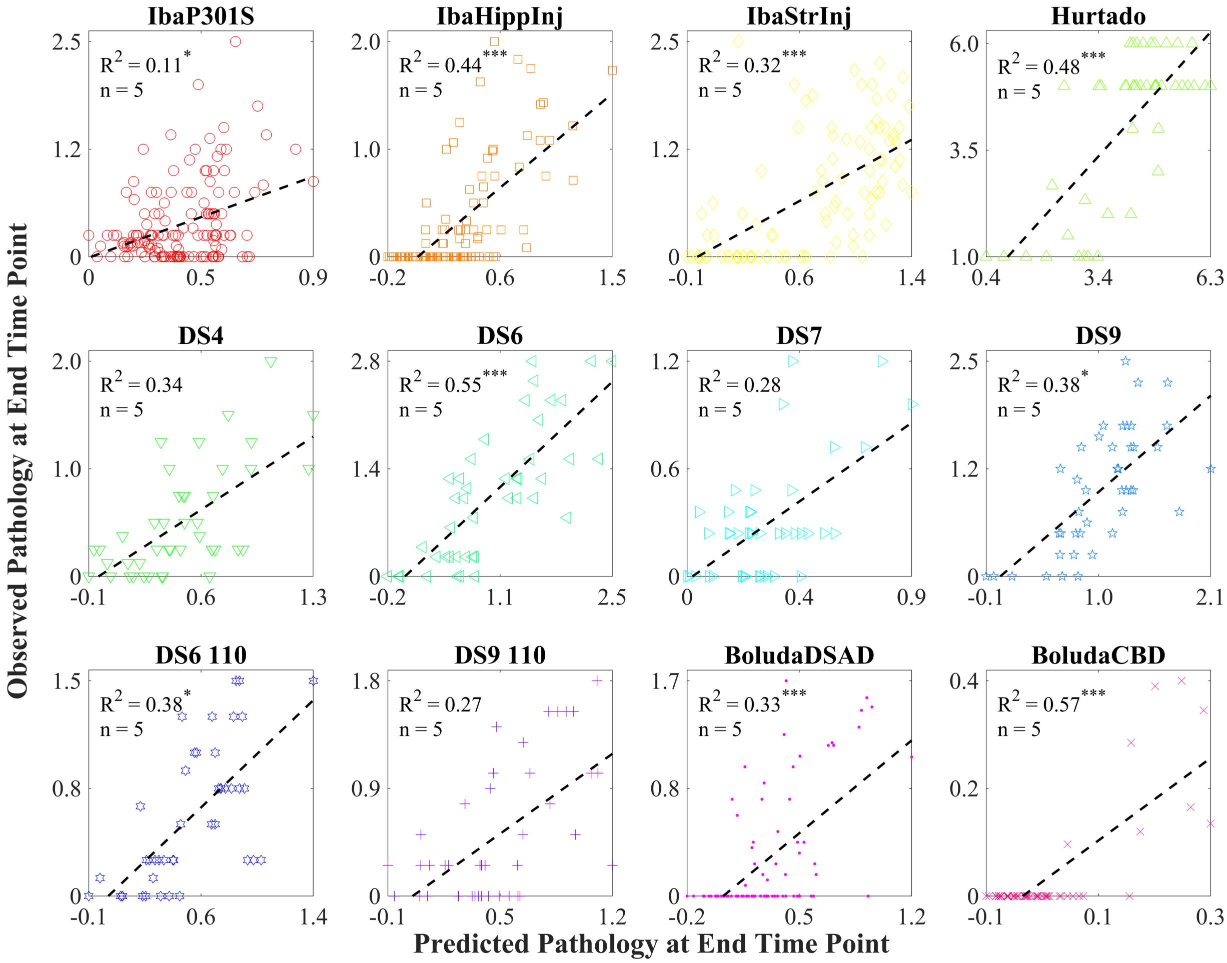

Figure S13: Multivariate analysis of end-timepoint pathology, AD genes. Scatter plots of the optimal cell-type-based models of tau pathology at the end time points for each of the nine mouse tauopathy studies, along with their associated R^2 values and the 5 genes with the highest correlations to pathology.

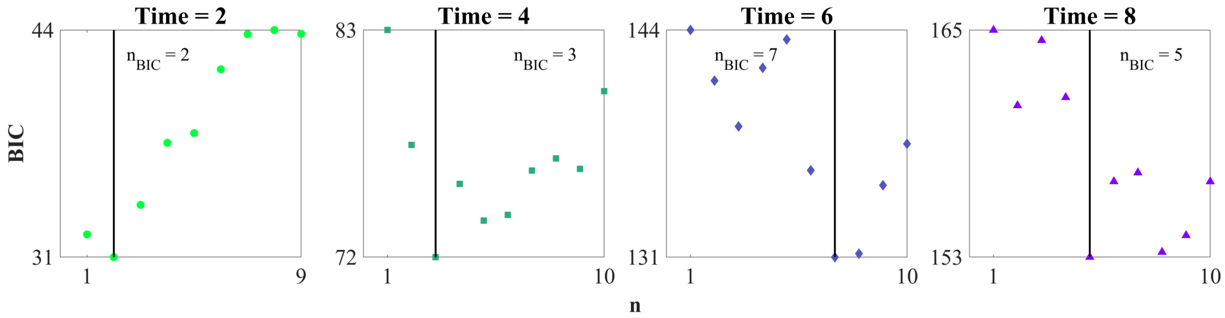

Figure S14: BIC plots for the multivariate linear models in Figure 4. Scatter plots of the BIC criterion with respect to the number of cell types added to the model (n) to determine the optimal sets the Hurtado, *et al.* dataset [4] for each timepoint.

Supplemental Tables

**Cortical glutamatergic neurons**		**Hippocampal glutamatergic neurons**	
Abbreviation	*Full name*	*Abbreviation*	*Full name*
Car3	*Car3*-expressing	CA1-ProS	CA1/prosubiculum
L2/3 IT CTX	Layer-2/3 intratelencephalic	CA2-FC-IG	CA2/fasciola cinereal/ induseum griseum
L4 RSP-ACA	Layer-4 retrospenial/ anterior cingulate	CA3	CA3
L4/5 IT CTX	Layer-4/5 intratelencephalic	CT SUB	Corticothalamic subiculum
L5 IT CTX	Layer-5 intratelencephalic	DG	Dentate gyrus
L5 PT CTX	Layer-5 pyramidal tract	L2 IT ENTl	Layer-2 intratelencephalic lateral entorhinal cortex
L5/6 NP CTX	Layer-5/6 near-projecting	L2 IT ENTm	Layer-2 intratelencephalic medial entorhinal cortex
L6 CT CTX	Layer-6 corticothalamic	L2/3 IT ENTl	Layer-2/3 intratelencephalic lateral entorhinal cortex
L6 IT CTX	Layer-6 intratelencephalic	L2/3 IT PPP	Layer-2/3 intratelencephalic para/post/presubiculum
L6b CTX	Layer-6b	L2/3 IT RHP	Layer-2/3 intratelencephalic retrohippocampal
		L3 IT ENT	Layer-3 intratelencephalic entorhinal cortex
		L5 PPP	Layer-5 para/post/presubiculum
		L5/6 IT TPE-ENT	Layer-5/6 intratelencephalic temporal association/perirhinal/ ectorhinal/entorhinal
		L6 IT ENTl	Layer-6 intratelencephalic lateral entorhinal cortex
		L6b/CT ENT	Layer-6b/corticothalamic entorhinal cortex
		NP PPP	Near-projecting para/post/ presubiculum
		NP SUB	Near-projecting subiculum
		SUB-ProS	Subiculum/prosubiculum

Table S1: Glutamatergic cell types. List of the abbreviations and names of the glutamatergic cell types used in this study, each of which corresponds to a taxonomic subclass annotated by Yao *et al.* [1]. We have delineated these subclasses as being either cortical or hippocampal based on the annotations of their lower-level clusters.

GABAergic neurons		Non-neuronal cells	
Abbreviation	*Full name*	*Abbreviation*	*Full name*
CR	Cajal-Retzius	Astro	Astrocytes
Lamp5	*Lamp5*-expressing	Endo	Endothelial
Meis2	*Meis2*-expressing	Micro-PVM	Microglia/perivascular macrophages
Pvalb	*Pvalb*-expressing	Oligo	Oligodendrocytes
Sncg	*Sncg*-expressing	SMC-Peri	Smooth muscle cells/pericytes
Sst	*Sst*-expressing	VLMC	Vascular and leptomeningeal cells
Sst Chodl	*Sst*-expressing, long-range		
Vip	*Vip*-expressing		

Table S2: GABAergic and non-neuronal cell types. List of the abbreviations and names of the GABAergic and non-neuronal cell types used in this study, each of which corresponds to a taxonomic subclass annotated by Yao *et al.* [1]. At the level of subclasses, these types are not uniquely defined between cortical and hippocampal regions.

**Name**	**Model**	**Injection site**	**Injectate**	$\mathbf{n_{ROI}}$
IbaP301S [6]	PS19	Locus coeruleus (RH)	Synthetic PFFs from 2N4R P301S τ (T40/PS)	148
IbaHippInj [5]	PS19	Dentate gyrus (RH)	Synthetic PFFs from 2N4R P301S τ (T40/PS) and from truncated P301L τ (K18/PL)	102
IbaStrInj [5]	PS19	Caudoputamen (RH) and primary motor area (RH)	Synthetic PFFs from 2N4R P301S τ (T40/PS) and from truncated P301L τ (K18/PL)	96
Hurtado [4]	PS19/ PDAPP	None	None	45
DS4 [7]	PS19	CA1 (LH)	Isolated from AD brain homogenate; prominent nuclear inclusions ("speckles")	44
DS6 [7]	PS19	CA1 (LH)	Isolated from P301S mouse brain homogenate; fibril-like cytoplasmic inclusions ("threads")	44
DS7 [7]	PS19	CA1 (LH)	Recombinant fibrils; prominent nuclear inclusions ("speckles")	44
DS9 [7]	PS19	CA1 (LH)	Recombinant fibrils; prominent nuclear inclusions ("speckles")	44
DS6 110 [7]	PS19	CA1 (LH)	DS6 strain, 1:10 dilution	44
DS9 110 [7]	PS19	CA1 (LH)	DS9 strain, 1:10 dilution	44
BoludaDSAD [3]	PS19	CA1 (LH) and primary somatosensory area (LH)	DSAD brain homogenate	90
BoludaCBD [3]	PS19	CA1 (LH) and primary somatosensory area (LH)	CBD brain homogenate	58

Table S3: Mouse tauopathy datasets. List of the tauopathy datasets explored here with descriptions of four key experimental features: mouse genetic background, injection site, type of τ injected, and the number of regions for which τ pathology was quantified. All studies quantified τ pathology within hemispheres ipsilateral and contralateral to the injection site separately with the exception of Hurtado, which was bilaterally averaged. RH – right hemisphere; LH – left hemisphere; PFF – preformed fibrils; DSAD – Down Syndrome Alzheimer's disease; CBD – corticobasal dengeneration.

**One-way t-test**	**t-statistic**	$p_{\mathbf{MHC}}$	**Two-way t-test**	**t-statistic**	$p_{\mathbf{MHC}}$
Cortical glutamatergic	-5.20	3.32×10^{-6}	*Cortical glut. – Hippocampal glut.*	-7.72	8.07×10^{-13}
Hippocampal glut.	6.79	4.27×10^{-10}	*Cortical glut. – GABAergic*	0.320	1
GABAergic	-3.32	5.09×10^{-3}	*Cortical glut. – Non-neuronal*	-1.49	0.827
Non-neuronal	-0.964	1	*Hippocampal glut. – GABAergic*	6.60	1.79×10^{-10}
			Hippocampal glut. – Non-neuronal	4.19	3.79×10^{-5}
			GABAergic – Non-neuronal	-1.37	1

Table S4: T-test results for cell-type classes. Summary of one-way and two-way t-test results for distributions of Pearson's R values of the four cell-type classes within the Yao, *et al.* dataset: cortical glutamatergic neurons, hippocampal glutamatergic neurons, GABAergic neurons, and non-neuronal cells (see **Figure 2C**). T-tests were performed after first using the Fisher's R-to-Z transformation on the individual Pearson's R values displayed in **Figure 2A**. The p-values reported have been multiple-hypothesis corrected using the Bonferroni criterion. See **Tables S1** and **S2** for a complete list of the cell types within each class.

Gene symbol	Gene name	Biological function
Adamts1	ADAM metallopeptidase with thrombospondin type 1 motif 1	Extracellular matrix organization
Ank3	Ankyrin-3	Membrane-cytoskeleton linker
Apoe	Apolipoprotein E	Negative regulation of apoptotic process
App	Amyloid-beta precursor protein	Axonogenesis, neurite growth, neuronal adhesion
Bace1	Beta-secretase 1	Proteolysis of amyloid-beta precursor protein
Cd33	Myeloid cell surface antigen CD33	Cell adhesion, cell-cell interactions
Clu	Clusterin	Extracellular chaperone protein
Doc2a	Double C2-like domain-containing protein alpha	Ca^{2+}-dependent neurotransmitter release
Epdr1	Mammalian ependymin-related protein 1	Cell-matrix adhesion
Grid2	Glutamate receptor ionotropic, delta-2	Glutamate receptor
Grin2b	Glutamate receptor ionotropic, NMDA 2B	Glutamate receptor
Hs3st2	Heparan sulfate glucosamine 3-O-sulfotransferase 2	Glycosaminoglycan biosynthetic process
Il34	Interleukin-34	Proliferation, survival and differentiation of monocytes and macrophages
Mapk14	Mitogen-activated protein kinase 14	MAP kinase signalling pathway
Mapt	Microtubule-associated protein tau	Microtubule assembly and stabilization
Pld3	5'-3' exonuclease PLD3	Regulates inflammtory response to single-stranded DNA
Prnp	Major prion protein	Unclear primary biological function
Rorb	Nuclear receptor ROR-beta	DNA-binding transcription factor
Sirpa	Tyrosine-protein phosphatase non-receptor type substrate 1	Cell surface receptor, cell adhesion
Slc44a1	Choline transporter-like protein 1	Choline transporter
Sorl1	Protein Sortilin-related receptor	Intracellular protein trafficking and localization
Spp1	Osteopontin	Cell-matrix adhesion
Tmem41a	Transmembrane protein 41A	Unclear primary biological function
Trem2	Triggering receptor expressed on myeloid cells 2	Disease-associated microglia activation

Table S5: AD-associated genes. List of the names and brief descriptions of the genes examined using univariate (**Figure 4**) and multivariate (**Figure S11** and **Figure S13**) selective vulnerability analyses, each of which has one or more variants associated with AD incidence. These genes represent an intersection between the list given by the Alzheimer's Disease Sequencing Project [8, 9] and the coronal series of the Allen Gene Expression Atlas (AGEA) [10], which yielded 24 genes. Gene annotations were obtained from the UniProt database [11] unless otherwise noted.

**Dataset**	**Cell types (top 5)**			**AD risk genes (top 5)**		
	R^2	n	BIC	R^2	n	BIC
IbaP301S [6]	**0.13***	**5**	**192.7**	0.11*	5	196.6
IbaHippInj [5]	**0.54*****	**5**	**93.4**	0.44***	5	114.1
IbaStrInj [5]	**0.43*****	**5**	**170.4**	0.32***	5	193.2
Hurtado [4]	**0.64*****	**5**	**153.3**	0.48***	5	169.0
DS4 [7]	**0.43****	**5**	**54.5**	0.34	5	61.2
DS6 [7]	**0.65*****	**5**	**79.4**	0.55***	5	90.2
DS7 [7]	**0.79*****	**5**	**-23.5**	0.28	5	29.9
DS9 [7]	**0.45****	**5**	**79.4**	0.38*	5	84.3
DS6 110 [7]	**0.46****	**5**	**48.7**	0.38*	5	54.3
DS9 110 [7]	**0.46****	**5**	**60.0**	0.27	5	72.8
BoludaDSAD [3]	**0.53*****	**5**	**68.7**	0.33***	5	99.4
BoludaCBD [3]	**0.78*****	**5**	**-163.9**	0.57***	5	-128.1

Table S6: Top-five feature linear model statistics. Statistics corresponding to the linear models shown in **Figure S10** and **Figure S13**. Bold font indicates the best model by the Bayesian Information Criterion (BIC). *: $p < 0.01$; **: $p < 0.001$; ***: $p < 0.0001$.

t (months)	R^2	n	BIC	Most significant cell type	t-statistic	p-value
2	0.28^{*}	2	30.7	L3 IT ENT	2.9	6.7×10^{-3}
4	0.52^{***}	3	71.2	Sst	3.7	6.2×10^{-4}
6	0.69^{***}	7	131.2	CT SUB	3.3	2.1×10^{-3}
8	0.63^{***}	5	153.3	Oligo	-3.4	1.5×10^{-3}

Table S7: Hurtado dataset linear model statistics. Statistics corresponding to the linear models shown in **Figure 5A**, along with the cell type with the coefficient with the single-highest t-statistic. *: $p < 0.01$; **: $p < 0.001$; ***: $p < 0.0001$.

Table S8-S11. Gene lists for SV-G, SV-C, SR-G, and SR-C gene ontology analysis.

References

[1] Yao, Z. *et al.* A taxonomy of transcriptomic cell types across the isocortex and hippocampal formation. *Cell* **184**, 3222–3241.e26 (2021). URL https://linkinghub.elsevier.com/retrieve/pii/S0092867421005018.

[2] Mezias, C., Torok, J., Maia, P. D., Markley, E. & Raj, A. Matrix Inversion and Subset Selection (MISS): A pipeline for mapping of diverse cell types across the murine brain. *Proceedings of the National Academy of Sciences* **119**, e2111786119 (2022). URL https://pnas.org/doi/full/10.1073/pnas.2111786119.

[3] Boluda, S. *et al.* Differential induction and spread of tau pathology in young PS19 tau transgenic mice following intracerebral injections of pathological tau from Alzheimer's disease or corticobasal degeneration brains. *Acta Neuropathologica* **129**, 221–237 (2015). URL http://link.springer.com/10.1007/s00401-014-1373-0.

[4] Hurtado, D. E. *et al.* Aβ Accelerates the Spatiotemporal Progression of Tau Pathology and Augments Tau Amyloidosis in an Alzheimer Mouse Model. *The American Journal of Pathology* **177**, 1977–1988 (2010). URL https://linkinghub.elsevier.com/retrieve/pii/S0002944010602489.

[5] Iba, M. *et al.* Synthetic Tau Fibrils Mediate Transmission of Neurofibrillary Tangles in a Transgenic Mouse Model of Alzheimer's-Like Tauopathy. *Journal of Neuroscience* **33**, 1024–1037 (2013). URL https://www.jneurosci.org/lookup/doi/10.1523/JNEUROSCI.2642-12.2013.

[6] Iba, M. *et al.* Tau pathology spread in PS19 tau transgenic mice following locus coeruleus (LC) injections of synthetic tau fibrils is determined by the LC's afferent and efferent connections. *Acta Neuropathologica* **130**, 349–362 (2015). URL http://link.springer.com/10.1007/s00401-015-1458-4.

[7] Kaufman, S. K. *et al.* Tau Prion Strains Dictate Patterns of Cell Pathology, Progression Rate, and Regional Vulnerability In Vivo. *Neuron* **92**, 796–812 (2016). URL https://linkinghub.elsevier.com/retrieve/pii/S0896627316306973.

[8] Bellenguez, C. *et al.* New insights into the genetic etiology of Alzheimer's disease and related dementias. *Nature Genetics* **54**, 412–436 (2022). URL https://www.nature.com/articles/s41588-022-01024-z.

[9] Kunkle, B. W. *et al.* Genetic meta-analysis of diagnosed Alzheimer's disease identifies new risk loci and implicates Aβ, tau, immunity and lipid processing. *Nature Genetics* **51**, 414–430 (2019). URL http://www.nature.com/articles/s41588-019-0358-2.

[10] Lein, E. S. *et al.* Genome-wide atlas of gene expression in the adult mouse brain. *Nature* **445**, 168–176 (2007). URL http://www.nature.com/articles/nature05453.

[11] Bateman, A. *et al.* UniProt: the Universal Protein Knowledgebase in 2023. *Nucleic Acids Research* **51**, D523–D531 (2023). URL https://academic.oup.com/nar/article/51/D1/D523/6835362.
